# p-21 activated kinase 4 promotes proliferation and survival of pancreatic cancer cells through AKT- and ERK-dependent activation of NF-κB pathway

**DOI:** 10.18632/oncotarget.2398

**Published:** 2014-08-27

**Authors:** Nikhil Tyagi, Arun Bhardwaj, Ajay P. Singh, Steven McClellan, James E. Carter, Seema Singh

**Affiliations:** ^1^ Department of Oncologic Sciences, Mitchell Cancer Institute, University of South Alabama, Mobile, Alabama, USA; ^2^ Department of Biochemistry and Molecular Biology, College of Medicine, University of South Alabama, Mobile, Alabama, USA; ^3^ Department of Pathology, College of Medicine, University of South Alabama, Mobile, Alabama, USA

**Keywords:** PAK4, pancreatic cancer, cell proliferation, apoptosis, Akt, ERK, NF-κB

## Abstract

Identification of novel molecular targets and understanding the mechanisms underlying the aggressive nature of pancreatic cancer (PC) remain prime focus areas of research. Here, we investigated the expression and pathobiological significance of p21-activated kinase 4 (*PAK4*), a gene that was earlier shown to be amplified in a sub-set of PC. Our data demonstrate PAK4 overexpression in PC tissues and cell lines with little or no expression in the normal pancreas. PAK4 silencing in two PC cell lines, MiaPaCa and T3M4, by RNA interference causes suppression of growth and clonogenic ability due to decreased cell cycle progression and apoptosis-resistance. PAK4-silenced PC cells exhibit altered expression of proliferation- and survival-associated proteins. Moreover, we observe decreased nuclear accumulation and transcriptional activity of NF-κB in PAK4-silenced PC cells associated with stabilization of its inhibitory protein, IκBα. Transfection of PAK4-silenced PC cells with constitutively-active mutant of IKKβ, an upstream kinase of IκBα, leads to restoration of NF-κB activity and PC cell growth. Furthermore, we show that PAK4-induced NF-κB activity is mediated through activation and concerted action of ERK and Akt kinases. Together, these findings suggest that PAK4 is a regulator of NF-κB pathway in PC cells and can serve as a novel target for therapy.

## INTRODUCTION

Pancreatic cancer (PC) is one of the most lethal malignancies in the United States [1]. According to an estimate by American Cancer Society, nearly 46,420 Americans will be diagnosed with PC in 2014 and approximately 39,590 will die due to this devastating disease, marking it as the fourth leading cause of cancer-related death [1]. The overall median post-diagnosis survival of PC patients is ~2-8 months, and 5 years survival rate is only about 6 % underscoring its impact on human health and wellbeing [1, 2]. Clearly, there is a critical need to develop effective treatment strategies to tackle this deadly malignancy.

The p21-activated kinases (PAKs) belonging to the serine/threonine protein kinases family are considered as major downstream effector molecules of Cdc42 and Rac1, the key small Rho GTPase proteins [3-5]. Besides this, recent evidence suggests that PAKs can also be activated by Rho GTPase-independent signals [6, 7]. Initially, PAKs were identified as cell morphology regulatory proteins [3], but later their important roles in other cellular processes including proliferation, survival, motility, etc. have also been reported [8-10]. On the basis of their structure and regulatory functions, PAKs have been broadly classified into two groups: group I (PAK 1-3) and group II (PAK 4-6) [5]. Among the group II PAKs, PAK4 is the most extensively and profoundly studied member. *PAK4* has been shown to be highly expressed in embryonic stage, whereas its low expression is observed in majority of the normal adult tissues suggesting its importance in the embryo development [11]. An overexpression of *PAK4* has also been observed in tumor tissues and cell lines of various origins [12, 13]. Moreover, an association of high *PAK4* expression with the poor prognosis in ovarian cancer patients has been demonstrated [14]. Besides this, a functional association of PAK4 with tumor phenotypes has also been established in some cancers [14-17].

*PAK4* gene is localized at chromosome 19q13, a region amplified in pancreatic tumor specimens [18]. However, till date, there is no report on its pathological significance in PC. In the present study, we investigated *PAK4* expression and function in PC by conducting a series of *in vitro* functional assays. Our data demonstrate that *PAK4* expression is associated with increased growth of PC cells resulting from enhanced cell-cycle progression and apoptosis-resistance. Moreover, mechanistic studies reveal the involvement of Akt- and ERK-mediated activation of NF-κB signaling in PAK4-induced growth of PC cells. Together, these findings provide first experimental evidence for a functional role of PAK4 in PC and suggest that it could serve as a novel target for PC therapy.

## RESULTS

### PAK4 is overexpressed in pancreatic cancer

**Figure 1:**
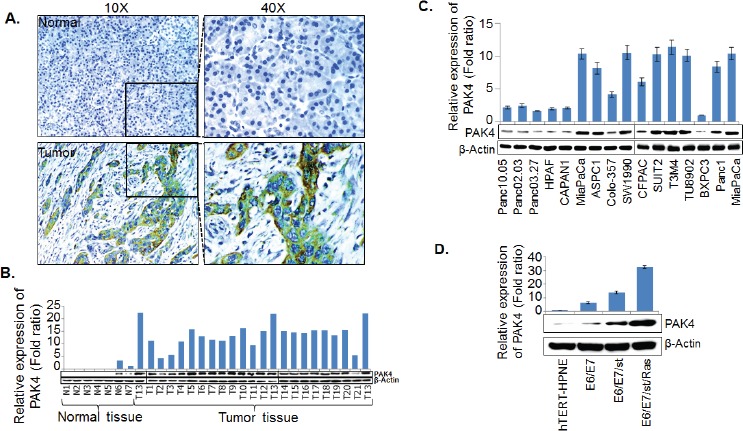
PAK4 expression analysis in pancreatic cancer tissue specimens and cell lines (A) Immunohistochemical assay was performed on paraffin embedded tissue microarray containing tissue sections (in triplicates) of normal pancreas (n=9) and pancreatic tumor (n=56) using PAK4 specific antibody. PAK4 expression was examined by immunoblot assays in (B) frozen pancreatic tissues [normal (n=7) and malignant n=21] specimens, (C) PC cell lines and (D) *in vitro* pancreatic cancer progression model (hTERT-HPNE and derived cell lines). β-actin was used as internal control. Bars (mean ± SEM, n=3) indicate the normalized expression levels of PAK4.

To investigate the clinical significance of PAK4 in PC pathobiology, we first examined its expression in normal pancreas (n=9) and PC tissue specimens (n=56) by IHC assay. Data demonstrate that ~96.4 % of the total tumor samples have an intense staining of PAK4, which is predominantly localized in the cytoplasm with some diffuse staining in the nucleus. However, no staining of PAK4 was observed in normal pancreatic tissues (Figure 1A). In the group of PAK4-positive tumor specimens, 25 (44.6 %) were weakly stained, 19 (33.9 %) were moderately stained and the remaining 10 (17.9 %) tumor specimens were strongly stained (Table 1). In addition, PAK4 expression was also examined in frozen tissue samples of PC (n=21) along with normal pancreatic tissues (n=7) by immunoblot analysis. Data show an overexpression of PAK4 in all the PC tissues, while no expression is observed in 5 normal tissues, while two are weakly positive (Figure 1B). Furthermore, PAK4 expression was assessed in a panel of established PC cell lines having varying tumorigenic and metastatic potential [19]. Data demonstrate a differential expression pattern of PAK4 in PC cell lines (Figure 1C). Next, we also examined the expression of PAK4 in pancreatic cancer progression (hTERT-HPNE and derived cell lines) model to correlate the expression of PAK4 with progression of pancreatic cancer. We observed gradually increased expression of PAK4 in this model (Figure 1D). Together, these findings confirm an overexpression of PAK4 in PC.

**Table 1:** PAK4 expression in normal and pancreatic tumor tissue specimens

Samples		Intensity	Total number (%)	Mean composite score ± SEM
Normal (n=9)	Negative	0	9 (100 %)	0
Tumor (n=56)	Negative	0	2 (3.6 %)	5.22 ± 0.29
	Positive	1+	25 (44.6 %)	
		2+	19 (33.9 %)	
		3+	10 (17.9 %)	

### Silencing of PAK4 decreases growth and clonogenic potential of pancreatic cancer cells

**Figure 2:**
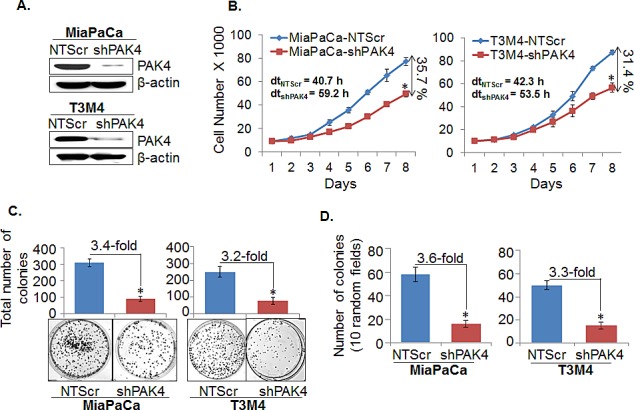
PAK4-overexpression is associated with increased growth and clonogenic potential of pancreatic cancer cells (A) Total protein from the stable pooled population of PAK4-silenced (MiaPaCa-shPAK4 and T3M4-shPAK4) along with their respective control cells (MiaPaCa-NTScr and T3M4-NTScr) was isolated and PAK4 expression was determined by immunoblot analyses. β-actin was used as loading control. (B) Cells (1×10^4^) were seeded in 6-well plates; growth was monitored by counting the cell number daily up to 8 days. The doubling time (dt) and percent inhibition in growth on 8^th^ day was calculated as described in Materials & Methods. (C) For anchorage dependent clonogenicity assay, cells were seeded at low density (1×10^3^cells/well) in regular media. After 2 weeks, colonies were stained with crystal violet, visualized, photographed and counted using imaging system. (D) For anchorage independent clonogenicity assay, cells (2.5×10^3^ cells/mL) suspended in regular media containing 0.4 % agarose were seeded in 6-well plate having bottom layer of 0.8 % agar growth medium and allowed to form colonies for 3 weeks. After 3 weeks, colonies were visualized and counted using Nikon eclipse microscope. Data represent as mean ± SEM. n=3, *, p< 0.05.

To gain insight into the pathobiological involvement of PAK4 in PC, we silenced its expression in two high PAK4 expressing, tumorigenic and aggressive cell lines, MiaPaCa and T3M4, by stable transfection of PAK4-targeted shRNA (shPAK4) or non-targeted scrambled sequence (NTScr) expression constructs. Stable transfectants were selected in antibiotic-selection media and the expression of PAK4 was analyzed by immunoblot assay. The clones that exhibited efficient downregulation of PAK4 were pooled for further analyses. Data show that the pooled population of PAK4-silenced clones exhibit significant knockdown of PAK4 in both MiaPaCa-shPAK4 and T3M4-shPAK4 cells as compared to their respective controls (MiaPaCa-NTScr and T3M4-NTScr) (Figure 2A). We next performed *in vitro* assays to examine the effects of PAK4-silencing on the growth characteristics and clonogenic ability of PC cells. Our data from growth kinetic assay demonstrate that the growth rate of PAK4-silenced (MiaPaCa-shPAK4 and T3M4-shPAK4) PC cells is significantly lower as compared to that of the respective control (MiaPaCa-NTScr and T3M4-NTScr) cells (Figure 2B). The growth of MiaPaCa-shPAK4 and T3M4-shPAK4 is decreased by ~35.7 % and 31.4 %, respectively, on 8^th^ day of culture in comparison with their respective controls (Figure 2B). The population doubling time (dt) calculated during exponential growth phase is increased from 40.7 h to 59.2 h and from 42.3 h to 53.5 h upon PAK4 silencing in MiaPaCa and T3M4 cells, respectively (Figure 2B). Furthermore, PAK4 silenced (MiaPaCa-shPAK4 and T3M4-shPAK4) cells show diminished plating efficiency (3.4 and 3.2 folds, respectively) compared to their respective control cells (Figure 2C). In anchorage-independent colony forming assay, we observe that the clonogenic potential of MiaPaCa-shPAK4 and T3M4-shPAK4 cells is decreased ~3.6 and 3.3 folds in comparison with MiaPaCa-NTScr and T3M4-NTScr cells, respectively (Figure 2D). Taken together, our findings suggest that PAK4 plays an important role in growth promotion of PC cells.

### PAK4 promotes cell cycle progression and apoptosis resistance

**Figure 3:**
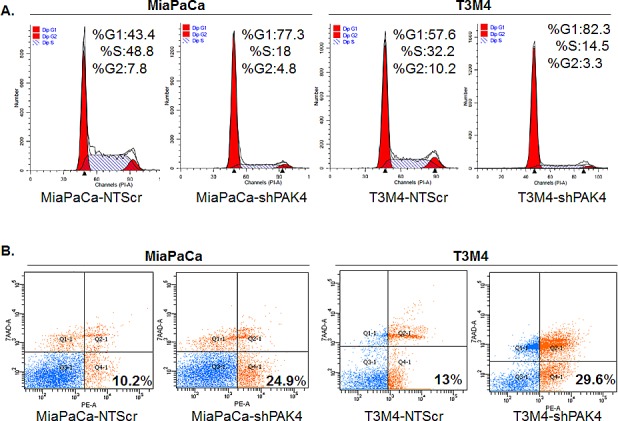
PAK4 facilitates cell cycle progression and confers apoptosis resistance (A) For cell cycle analysis, PAK4 silenced (MiaPaCa-shPAK4 and T3M4-shPAK4) cells along with their respective controls (MiaPaCa-NTScr and T3M4-NTScr) were synchronized by culturing them in serum-free media for 72 h, and then incubated in regular medium for 24 h. Subsequently, distribution of cells in different phases of cell cycle was analyzed by Propidium iodide (PI) staining followed by flow cytometry. (B) For apoptosis assay, PC cells were grown in regular media for 72 h and percentage of apoptotic cells was analyzed by PE Annexin V and 7AAD staining followed by flow cytometry.

**Figure 4:**
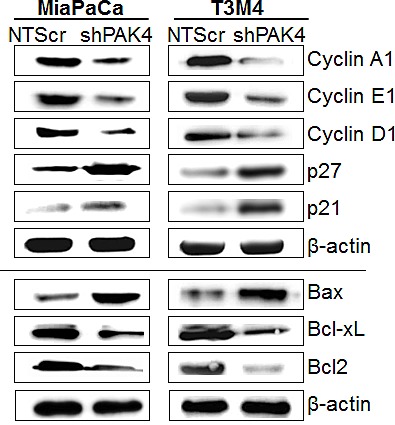
Effect of PAK4 silencing on the expression of proteins associated with cell-cycle and apoptosis Total protein from PAK4-silenced (MiaPaCa-shPAK4 and T3M4-shPAK4) cells along with their scrambled control (MiaPaCa-NTScr and T3M4-NTScr) was isolated and expression of various cell cycle and survival-associated proteins was examined by immunoblot analysis. β-actin was used as an internal control.

In general, an enhanced growth of a cancer cell is a cumulative result of increased cell cycle progression and apoptosis resistance [20]. Therefore, flow-cytometry based assays were executed to investigate the role of PAK4 in cell cycle progression and apoptosis resistance of PC cells. Our cell cycle analysis data indicate that the PAK4 silencing causes a G1 phase cell cycle arrest in PC cells, which is clearly evident by the more number of MiaPaCa-shPAK4 and T3M4-shPAK4 cells in G1-phase (77.3 % and 82.3 %, respectively) as compared to MiaPaCa-NTScr (43.4 %) and T3M4-NTScr (57.6 %) cells (Figure 3A). Moreover, apoptosis assay results show that the PAK4-silenced (MiaPaCa-shPAK4 and T3M4-shPAK4) cells are more apoptotic (~2.4 and 2.3 folds, respectively) as compared to their controls (Figure 3B). After confirming the role of PAK4 in cell cycle progression and apoptotic resistance, we next examined the expression profile of proteins associated with cell proliferation and survival by immunoblot assay in PAK4- silenced and control cells to decipher the mechanistic basis of these effects. We observed that MiaPaCa-shPAK4 and T3M4-shPAK4 cells have decreased expression of cyclins (A1, D1 and E1) and anti-apoptotic proteins (Bcl2 and Bcl-xL) as compared to MiaPaCa-NTScr and T3M4-NTScr cells, respectively. In contrast, high expression of p27 and p21 (Cyclin-dependent kinase inhibitors) and pro-apoptotic protein Bax in PAK4 silenced (MiaPaCa-shPAK4 and T3M4-shPAK4) cells is observed (Figure 4). Together, these findings indicate that the PAK4 enhances growth of PC cells by inducing cell cycle progression and apoptosis resistance.

### NF-κB is involved in PAK4-induced proliferation and survival of pancreatic cancer cells

**Figure 5:**
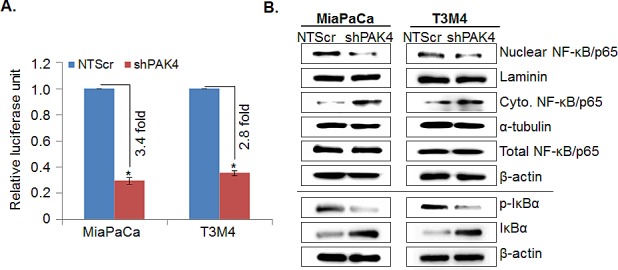
PAK4 induces transcriptional activity of NF-κB/p65 in human pancreatic cancer cells by promoting its nuclear translocation (A) Sub-confluence level of MiaPaCa and T3M4 cells were co-transfected with NF-κB responsive luciferase reporter and TK-Renilla luciferase (control) plasmids. 48 h post-transfection, cells were harvested in passive lysis buffer and luciferase (Fire-fly; test and Renilla, transfection efficiency control) activity was assessed using a dual-luciferase assay system. Data is presented as normalized fold-change in luciferase activity (mean± SEM; n = 3, *, p < 0.05). (B) Total, nuclear and cytoplasmic extracts were prepared and expression of NF-κB/p65, p-IκBα (S32/36) and IκBα was determined by immunoblot analysis. Laminin (for nuclear fraction), α-tubulin (for cytoplasmic fraction) and β-actin (for total fraction) were used as loading controls.

**Figure 6:**
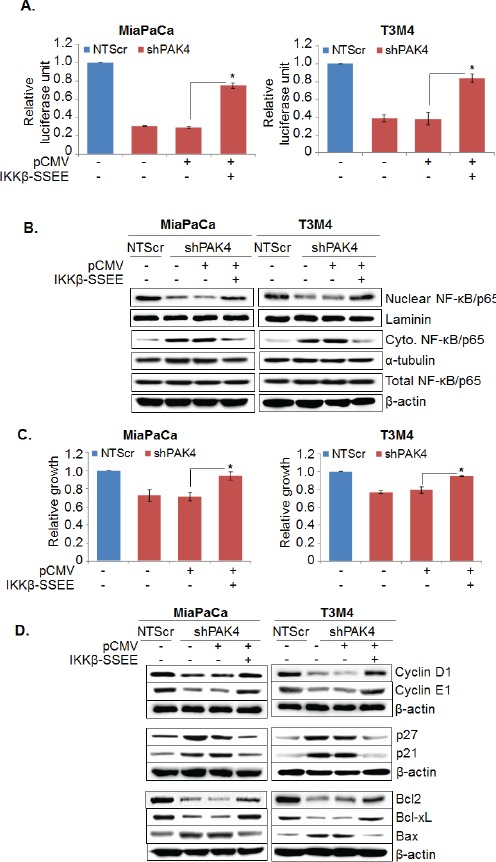
NF-κB-mediates PAK4-induced proliferation and apoptosis resistance in pancreatic cancer cells PAK4 silenced (MiaPaCa-shPAK4 and T3M4-shPAK4) cells were transfected with constitutively active IKKβ mutant (IKKβ-SSEE) or empty vector (pCMV). (A) After 48 h transfection, cells were again transfected with NF-κB -luciferase promoter-reporter constructs and NF-κB transcriptional activity was examined as described previously. Bars represent the mean of triplicates ± SEM, *, p < 0.05. (B) Nuclear and cytoplasmic fractions were prepared after 24 h of transfection and expression level of NF-κB was examined by immunoblot analysis. Laminin (for nuclear fraction), α-tubulin (for cytoplasmic fraction) and β-actin (for total fraction) were used as loading controls. (C) After 72 h of transfection, cell viability was examined by WST-1 assay. Bars represent the mean of triplicates ± SEM, *, p < 0.05. (D) Total protein lysate was prepared after 48 h of transfection and expression level of various cell cycle and survival-associated proteins were examined by immunoblot analysis. β-actin was used as loading control.

NF-κB is constitutively active in many cancers, including PC and it has been shown that NF-κB plays a significant role in facilitating cell proliferation and apoptosis resistance [21, 22]. Therefore, we next studied the involvement of NF-κB in PAK4-mediated pathobiological functions. The effect of PAK4 silencing on transcriptional activity of NF-κB was analyzed by luciferase-based promoter reporter assay. As shown in Figure 5A, the transcriptional activity of NF-κB responsive promoter was decreased by ~3.4 fold in MiaPaCa-shPAK4 and ~2.8 fold in T3M4-shPAK4 as compared to that in control cells. These findings were further confirmed by immunoblot analysis. Our data show a decreased nuclear accumulation of NF-κB/p65 that correlates with its increased cytoplasmic levels in PAK4-silenced (MiaPaCa-shPAK4 and T3M4-shPAK4) cells as compared to their respective control (MiaPaCa-NTScr and T3M4-NTScr) cells (Figure 5B, upper panel). We also studied the effects of PAK4-silencing on IκBα, which inhibits NF-κB by keeping it sequestered into the cytoplasm [22]. Data demonstrate a significant decrease in the level of phospho-IκBα, while simultaneously increase in the level of total IκBα in PAK4-silenced (MiaPaCa-shPAK4 and T3M4-shPAK4) cells in comparison to PAK4 expressing (MiaPaCa-NTScr and T3M4-NTScr) cells (Figure 5B, lower panel). To confirm the role of NF-κB in PAK4-induced growth, PAK4-silenced cells were transfected with constitutively active mutant of IKKβ (IKKβ-SSEE) or control vector (pCMV). Thereafter, its effect on transcriptional activity of NF-κB, cell growth, and the proteins associated with cell proliferation and apoptosis was examined. Our data show that upon IKKβ mutant transfection, transcriptional activity and nuclear accumulation of NF-κB/p65 in PAK4-silenced cells was restored (Figure 6A & B), which correlated with regained cell growth (Figure 6C). Furthermore, transfection of constitutively active IKKβ mutant also restored the expression of cell cycle promoting and anti-apoptotic proteins in PAK4-silenced cells (Figure 6D). Together, these findings suggest that PAK4 enhances pancreatic cell growth by promoting nuclear accumulation and transcriptional activity of NF-κB.

### PAK4-enhances nuclear accumulation and transcriptional activity of NF-κB through activation of Akt and ERK pathways

**Figure 7:**
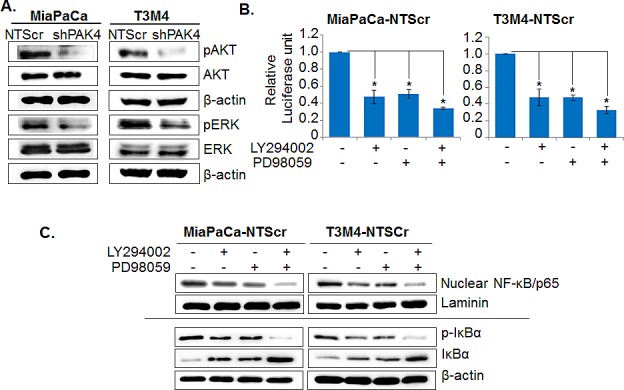
PAK4-activated Akt and ERK cooperatively promotes nuclear accumulation and transcriptional activity of NF-κB (A) Total cellular protein was isolated from PAK4-silenced (MiaPaCa-shPAK4 and T3M4-shPAK4) and control (MiaPaCa-NTScr and T3M4-NTScr) cells and expression level of Akt, p-Akt, ERK, p-ERK was examined by immunoblot analysis. β-actin was used as loading control. (B) PAK4 expressing (MiaPaCa-NTScr and T3M4-NTScr) cells were treated with Akt inhibitor (LY294002, 20 μm) or ERK inhibitor (PD98059, 25 μm) 1 h prior to the transfection of NF-κB-luciferase promoter-reporter construct and NF-κB transcriptional activity was examined after 48 h as described previously. Bars represent the mean of triplicates ± SEM, *, p< 0.05. (C) Total and nuclear protein extracts from the MiaPaCa-NTScr and T3M4-NTScr cells treated with Akt and/or ERK inhibitors alone or in combination for 24 h were prepared and effects on NF-κB/p65 (in nuclear), p-IκBα, and IκBα (in total) were examined by immunoblot analysis. Laminin (for nuclear fraction) and β-actin (for total fraction) were used as loading.

After confirming the role of NF-κB in PAK4-mediated PC cell growth, we next sought out to delineate the molecular mechanism underlying PAK4-induced NF-κB activation. For this, we primarily focused on two known downstream mediators of PAK4, Akt and ERK, which are also shown to regulate NF-κB in cancer cells [23-25]. Our immunoblot data demonstrate significantly decreased expression of phospho-Akt as well as phospho-ERK in PAK4 knockdown PC cells, while no noticeable effect is observed on the expression of total Akt and ERK (Figure 7A). To confirm the participation of Akt and ERK in PAK4-induced activation of NF-κB, MiaPaCa-NTScr and T3M4-NTScr cells were treated with pharmacological inhibitors of Akt (LY294002) and ERK (PD98059), alone or in combination, and their effects on the nuclear accumulation/transcriptional activity of NF-κB was examined. The data show a partial reduction in the transcriptional activity of NF-κB upon inhibition of Akt as well as ERK in both the PC cells, whereas their combined inhibition caused a more potent suppression (Figure 7B). A similar effect of the Akt and/or ERK inhibition on nuclear accumulation of NF-κB/p65 was observed in PC cells (Figure 7C, upper panel). Having observed association of cellular IκBα level with the PAK4-induced nuclear accumulation and transcriptional activity of NF-κB, we also examined the effects of Akt and/or ERK inhibition of the IκBα in PC cells by immunoblot assay. Our data show that inhibition of either Akt or ERK increases the expression level of IκBα in both the PC cells, which is associated with a concomitant decrease in its phosphorylation status (Figure 7C, lower panel). Therefore, these findings suggest a role of PAK4-mediated activation of Akt/ERK pathways in enhanced nuclear accumulation and transcriptional activity of NF-κB.

## DISCUSSION

The present study demonstrated, for the first time, a pathobiological role of PAK4 in proliferation and survival of PC cells. Moreover, the data revealed the participation of Akt- and ERK-mediated activation of NF-κB in PAK4-induced growth of PC cells. PAK4 has earlier been shown to be amplified in a sub-set of PC tissue [18]. Similarly, we also observed an overexpression of PAK4 in PC tissues and established cell lines, while it was not expressed or expressed at low levels in normal pancreatic tissues. It is; however, yet to be established whether PAK4 overexpression could solely be ascribed to gene amplification or it might also involve other gene regulatory mechanisms.

Besides PC, PAK4 overexpression has also been reported for several other tumor types including ovarian, breast, gastric and colon tumors suggesting its important roles in tumor development [5, 12, 14]. Indeed, our *in vitro* studies provide clear evidence for a growth promoting role of PAK4 in PC. Similar findings have also been reported in earlier published studies [14, 16, 17]. It has been shown that PAK4-overexpression is associated with the tumorigenic potential of human breast cancer cells in athymic mice [10]. Similarly, it was reported that PAK4 promoted gastric tumorigenesis [26]. In another important observation, an essential role of PAK4 in K-Ras driven proliferation and colony formation ability of colon cancer cells was also established [27]. Considering the fact that K-Ras is mutated in ~95 % cases of pancreatic tumors, PAK4 might also be involved in mediating and/or regulating constitutive K-Ras activation-driven downstream signaling pathways involved in cell proliferation, survival and therapy resistance [28, 29].

The uncontrolled and rapid growth of cancer cells is primarily attributed to the enhanced cell-cycle progression and development of apoptotic resistance [20]. Similar to the other cellular event, cell-cycle and apoptosis processes are tightly regulated by specific proteins such as cyclins and their inhibitors (in case of cell-cycle) and anti/pro-apoptotic proteins (for survival) [30, 31]. Our data revealed that suppressed growth in PAK4-knockdown PC cells resulted from decreased cell-cycle progression and induction of apoptosis. Moreover, we also observed that PAK4 silencing led to altered expression of several proteins associated with cell-cycle (Cyclin D1, A, p21 and p27) and apoptosis (Bax, Bcl2 and Bcl-xL). Similar to our findings, importance of PAK4 in the regulation of cell-cycle and development of apoptosis resistance has also been reported by other groups as well [32]. Nekrasova and Minden in 2011 reported the arrest of PAK4-deleted fibroblast cells in G1 phase of cell-cycle, which was associated with increased p21 and decreased Cyclin D1 expression in these cells [15]. Furthermore, it was also demonstrated that PAK4 prevented cancer cells from undergoing apoptotic cell death by inhibiting the activation of caspases-3/8/7 and subsequently leading to PARP1 cleavage [33].

Role of NF-κB, a transcription factor, in the pathobiology of several cancers has been very well documented [21, 22]. NF-κB induces the expression of a number of oncogenes involved in the regulation of multiple cellular process including cell proliferation and survival [34, 35]. Moreover, emerging evidence suggests that NF-κB is constitutively active in PC [36, 37]; however, the exact molecular mechanism(s) responsible for the activation of NF-κB in PC is not well understood. In this context, we investigated the role of PAK4 in the activation of NF-κB/p65 in PC cells and its involvement in PAK4-mediated effects. We observed decreased transcription activity/nuclear accumulation of NF-κB/p65 in PAK4 knockdown cells, which was associated with inhibition of IκB-α phosphorylation and concomitant increase in its expression. Usually, IκB-α binds with NK-κB/p65 and keeps it sequestered in the cytoplasm of a cell. Upon phosphorylation by upstream kinases such as IKK, IκB-α gets degraded that causes release and subsequent nuclear translocation of NF-κB/p65, which then induces gene expression [22, 34]. Similar to current study, we have also witnessed IκB-α-mediated negative regulation of NF-κB/p65 in prostate cancer cells [38]. Our additional data using constitutive active mutant of IKK, an upstream negative regulator of IκB-α provided evidence to support an important role of NF-κB in PAK4-induced growth of PC cells through regulation of proliferation- and survival-associated genes. These findings corroborate other findings where NF-κB has been shown to regulate cell-cycle and apoptosis in other cancer types [34, 39].

Based on the studies conducted so far, it has been suggested that PAK4 can activate several signaling pathways responsible for tumorigenesis [4, 5, 13, 26, 27]. Here, we observed that the phosphorylation status of Akt and ERK in PC cells was directly associated with PAK4 expression. Moreover, our data demonstrated that both Akt and ERK played an important role in mediating the effect of PAK4 on subcellular localization of NF-κB/p65 and its transcriptional activity. This is consistent with our recent finding, where we have delineated the cooperative involvement of Akt and ERK pathways in the activation of NF-κB/p65 [40]. In another report, ERK has been identified as a downstream effector pathway mediating PAK4-induced pro-survival effects [41]. In addition, a recent study suggested the role of PI3/Akt and ERK pathways in the PAK4-induced cisplatin resistance in gastric cancer cells [23]. Interestingly, a positive reciprocal association between PAK4 and PI3K/Akt, in which both of the molecules activates each other, has also been demonstrated [23]. However, exact molecular mechanism(s) involved in the PAK4-mediated regulation of Akt and ERK pathways are yet to be characterized. It is likely that PAK4 may indirectly impact ERK and Akt phosphorylation through regulation of c-Src and EGFR, which are their known upstream activators [14].

In summary, our findings have provided first experimental evidence to support that PAK4 is overexpressed in PC and promotes proliferation and survival of PC cells via activation of oncogenic signaling pathways. These data suggest that PAK4 could serve as a novel diagnostic biomarker and/or is a promising target for therapeutic intervention in pancreatic cancer.

## MATERIALS AND METHODS

### Cell lines, tissue specimens and human tissue microarray

The human PC cell lines were maintained as monolayer cultures in RPMI-1640 or Dulbecco's Modified Eagle Medium (DMEM) medium (Invitrogen, Carlsbad, CA) supplemented with 10 % fetal bovine serum (FBS) (Atlanta Biologicals, Lawrenceville, GA), penicillin (100 units/mL) and streptomycin (100 μg/mL) (Invitrogen) in a humidified atmosphere of 5 % CO_2_ at 37 °C. All the cell lines were tested intermittently and determined to be free from mycoplasma. Frozen pancreatic tissue specimens (normal and malignant) were obtained through Cooperative Human Tissue Network (CHTN) at the University of Alabama at Birmingham (UAB) under an Institutional Review Board (IRB)-approved protocol. Pancreatic tissue microarray (PA207) containing both normal and pancreatic adenocarcinoma cores were purchased from US Biomax, Inc. (Rockville, MD).

### Antibodies and plasmids

Anti -PAK4, -Bax, -Bcl2, -Cyclin D1, -p-IκB-α (Ser32/36), (rabbit polyclonal), -Bcl-xL, -NF-κB/p65, -ERK1/2 (rabbit monoclona1), -IκB-α, -p-ERK1/2 (mouse monoclonal) antibodies were purchased from Cell Signaling Technology (Beverly, MA). Antibodies against Akt and p-Akt (rabbit monoclonal) were from Epitomics (Burlingame, CA). Antibodies targeting Cyclin E1, p21, Laminin, α-tubulin (mouse monoclonal), p27, Cyclin A1 (rabbit polyclonal) and respective anti-mouse or anti-rabbit horseradish peroxidase (HRP)-conjugated secondary antibodies were procured from Santa Cruz Biotechnology (Santa Cruz, CA). Anti-PAK4 antibody (rabbit polyclonal; for immunohistochemistry) was purchased from Pierce Biotechnology (Rockford, IL). β-actin (mouse monoclonal) antibody was purchased from Sigma-Aldrich (St. Louis MO). Plasmids expressing PAK4-targeting (PAK4-shRNA-pGFP-V-RS) and non-targeted scrambled (NTScr-shRNA-pGFP-V-RS) short hairpin RNA sequence and empty vector (pCMV) were purchased from Origene (Rockville, MD). pGL4.32 [luc2P/NF-B-RE/Hygro] and pRL-TK plasmids were from Promega (Madison, WI). pCMV-IKKβ S177E S181E (plasmid number 11105) was from A. Rao Laboratory and procured through Addgene (Cambridge, MA).

### Transfections and treatments

For the generation of stable PAK4 knockdown cell lines, PAK4 overexpressing PC (MiaPaCa and T3M4) cells were transfected with PAK4-shRNA-pGFP-V-RS along with control plasmid (NTScr-shRNA-pGFP-V-RS) using X-tremeGENE HP DNA Transfection Reagent (Roche, Indianapolis, IN) as per the manufacturer's instructions. The cells expressing PAK4 shRNA (MiaPaCa-shPAK4 and T3M4-shPAK4) or non-target scrambled control (MiaPaCa-NTScr and T3M4-NTScr) were selected in media containing Puromycin (2 μg/ml) and were assessed for PAK4 expression by immunoblotting. The clones showing significant knockdown of PAK4 were pooled and expanded for further experiments. For dissecting the role of NF-κB/p65, PAK4 knockdown cells were transiently transfected with constitutively active IKKβ mutant (pCMV-IKKβ S177E S181E) along with its control vector (pCMV) using X-tremeGENE HP DNA Transfection Reagent. To elucidate the role of Akt and ERK, cells were treated with 20 μM LY294002 (Akt inhibitor) and 25 μM PD98059 (ERK inhibitor) (Cell Signaling Technology) alone or in combination as described in respective figure legend.

### Immunoblot analysis

Total protein from the PC cells and frozen tissue samples was isolated and estimated using DC Protein Assay Kit (Bio-Rad, Hercules, CA). Thereafter, protein samples (80-120 μg) were resolved by SDS-PAGE and subjected to immunoblot analysis as described earlier [25, 42] using specific antibodies against various proteins. β-actin, α-tubulin and Laminin were used as loading controls for total, cytoplasmic and nuclear proteins, respectively. All the primary antibodies were used at 1:1000 dilution except antibodies against p27, Cyclin A1, Akt and p-Akt (1:500), whereas all the secondary antibodies were used at 1:2000 dilution. β-actin was used at 1:20,000 dilution. The signal was detected using ECL plus Western Blotting substrate (Thermo Scientific, Logan, UT) and LAS-3000 image analyzer (Fuji Photo Film Co., Tokyo, Japan).

### Growth kinetics assay

MiaPaCa and T3M4 cells (1×10^4^/well) were seeded in 6-well plates and allowed to grow for 8 days. The growth rate was determined by counting the number of viable cells by dye exclusion method using Countess® Automated Cell Counter (Life technology™, Carlsbad, CA), every day for eight days. Cell population doubling time (*dt*) was calculated during exponential growth phase (96-144 h) using the following formula: dt = 0.693 t/ln (Nt/N0), where t is time (in h), Nt is the cell number at time t, and N0 is the cell number at initial time. The percent growth inhibition on 8^th^ day was calculated using the formula: 100-[(N_shPAK4_/N_NTScr_) X 100], where N_shPAK4_ and N_NTScr_ are the number of cells on 8^th^ day.

### Clonogenicity assay

For anchorage-independent clonogenicity assay, cells (2.5×10^3^) suspended in 0.4 % agar in regular medium were plated in 6-well plate on the top of 0.8 % agar layer and allowed to form colonies under normal culture conditions for 3 weeks. For anchorage-dependent clonogenicity assay, cells (1×10^3^) were seeded in 6-well plate and allowed to grow for 2 weeks under normal culture conditions. After respective incubation period colonies were fixed, stained, counted and photographed (100X) using Nikon Eclipse microscope (Nikon Instruments Inc. Melville, NY) and counted.

### Cell cycle analysis

Cells (1×10^6^ cells/well) were synchronized by culturing them in serum-free media for 72 h. Subsequently, cells were grown in regular medium for 24 h, washed, trypsinized and fixed with 70 % ethanol overnight at 4 °C. Post fixation cells were washed, stained with Propidium Iodide using PI/RNase kit (BD Bio Sciences, San Jose, CA) and analyzed by flow-cytometry on a BD-FACS Canto™ II (BD Bio Sciences). The percentage of cell population in various phases of cell cycle was calculated using Mod Fit LT software (Verity Software House, Topsham, ME).

### Apoptosis assay

Apoptosis assay was performed using PE Annexin V apoptosis detection kit (BD Biosciences). Briefly, cells (1×10^5^ cells/well) were seeded in 6-well plate and allowed to grow for 72 h. Thereafter, the harvested cells were washed, suspended in 1 mL DNA binding buffer, incubated with PE Annexin V and 7AAD (7-Amino-Actinomycin-d) in the dark for 30 min at room temperature and analyzed by flow cytometry.

### Nuclear and cytoplasmic fractionation

The cytoplasmic and nuclear extracts were prepared using the Nuclear Extract Kit (Active Motif, Carlsbad, CA) In brief, cells were washed following treatment with 1 mL ice-cold PBS/phosphatase inhibitors, lysed in 500 μL hypotonic buffer and centrifuged at 14,000 g for 30 s at 4 °C. After collecting supernatant (cytoplasmic fraction), pellets were re-suspended in 50 μL complete lysis buffer, incubated on ice for 30 min and then centrifuged at 14,000 g for 10 min at 4 °C. The supernatant (nuclear fraction) were stored at -80 °C.

### NF-κB transcriptional activity assay

PC cells were grown in 6-well plate and transiently transfected with 1 μg of NF-κB-luciferase-based promoter-reporter construct i.e. pGL4.32 [luc2P/NF-κB -RE/Hygro] and 0.5 μg of control reporter plasmid (pRL-TK) containing *Renillareniformis* luciferase gene downstream of the TK promoter. After 48 h of transfection, the cells were harvested in passive lysis buffer and luciferase activity was measured using the Dual Luciferase Assay System (Promega).

### Immunohistochemical analysis

Immunohistochemical (IHC) analysis was performed on formalin-fixed, paraffin-embedded pancreatic tissue microarray slide containing tissue from normal pancreas and tumor samples from PC patients. In brief, tissue specimens were deparaffinized using EZ-Dewax (Biogenex, Fremont, CA), rehydrated and incubated in Peroxidazed I (Biocare Medical, Concord, CA) for 30 min to block endogenous peroxidase activity. Thereafter, antigen retrieval was achieved by using decloaking Chamber (Biocare Medical) according to manufacturer's protocol. Subsequently, tissue sections were blocked for 10 min with Background Sniper (Biocare Medical) and incubated with PAK4 specific antibody (1:2500, rabbit polyclonal) for 60 min at room temperature. After that, sections were incubated at room temperature with recommended polymer and probe (Biocare Medical) according to manufacturer's protocol. Immunoreactivity was visualized by using DAB Chromogen followed by hematoxylin counterstain. Thereafter, microarray slide was examined and scored by a pathologist on a four point scale [staining intensity: (0 to 3+) and percentage of positive cells: (0 to 4)] and composite score was calculated by multiplying intensity score by extent score.

### Statistical analysis

All the experiments were performed at least three times, independently and all data are expressed as “mean ± SEM”. Wherever appropriate, the data were also subjected to unpaired two tailed Student's t-test. p< 0.05 was considered statistically significant.
